# Myxozoan polar tubules display structural and functional variation

**DOI:** 10.1186/s13071-016-1819-4

**Published:** 2016-10-14

**Authors:** Jonathan Ben-David, Stephen D. Atkinson, Yulia Pollak, Gilad Yossifon, Uri Shavit, Jerri L. Bartholomew, Tamar Lotan

**Affiliations:** 1 Department of Marine Biology, Leon H. Charney School of Marine Sciences, University of Haifa, Haifa, 31905 Israel; 2 Department of Microbiology, Oregon State University, Nash Hall 226, Corvallis, OR 97331 USA; 3 Electron Microscopy Unit, Faculty of Natural Sciences, University of Haifa, Haifa, 31905 Israel; 4 Faculty of Mechanical Engineering, Technion, Haifa, 32000 Israel; 5 Faculty of Civil and Environmental Engineering, Technion, Haifa, 32000 Israel

**Keywords:** Myxozoa, *Myxobolus*, Cnidaria, Polar capsule, Injection, Cnidocyst, Whirling disease

## Abstract

**Background:**

Myxozoa is a speciose group of endoparasitic cnidarians that can cause severe ecological and economic effects. Although highly reduced compared to free-living cnidarians, myxozoans have retained the phylum-defining stinging organelles, known as cnidae or polar capsules, which are essential to initiating host infection. To explore the adaptations of myxozoan polar capsules, we compared the structure, firing process and content release mechanism of polar tubules in myxospores of three *Myxobolus* species including *M. cerebralis*, the causative agent of whirling disease.

**Results:**

We found novel functions and morphologies in myxozoan polar tubules. High-speed video analysis of the firing process of capsules from the three *Myxobolus* species showed that all polar tubules rapidly extended and then contracted, an elasticity phenomenon that is unknown in free-living cnidarians. Interestingly, the duration of the tubule release differed among the three species by more than two orders of magnitude, ranging from 0.35 to 10 s. By dye-labeling the polar capsules prior to firing, we discovered that two of the species could release their entire capsule content, a delivery process not previously known from myxozoans. Having the role of content delivery and not simply anchoring suggests that cytotoxic or proteolytic compounds may be present in the capsule. Moreover, while free-living cnidarians inject most of the toxic content through the distal tip of the tubule, our video and ultrastructure analyses of the myxozoan tubules revealed patterns of double spirals of nodules and pores along parts of the tubules, and showed that the distal tip of the tubules was sealed. This helical pattern and distribution of openings may minimize the tubule mechanical weakness and improve resistance to the stress impose by firing. The finding that myxozoan tubule characteristics are very different from those of free-living cnidarians is suggestive of their adaptation to parasitic life.

**Conclusions:**

These findings show that myxozoan polar tubules have more functions than previously assumed, and provide insight into their evolution from free-living ancestors.

**Electronic supplementary material:**

The online version of this article (doi:10.1186/s13071-016-1819-4) contains supplementary material, which is available to authorized users.

## Background

Myxozoa is a large and wide-spread group of microscopic, obligate endoparasites that have recently been recognized as a sister group to Medusozoa within the phylum Cnidaria [1–6]. Myxozoans are estimated to have diverged from free-living cnidarians during the Cambrian era [7] and today are among the most common fish parasites [8]. Although many have established benign, asymptomatic relationships with their hosts, some species cause devastating diseases of both natural and farmed fish populations. These diseases, including proliferative kidney disease, enteromyxosis and whirling disease, have severe ecological and economic impacts [9–13]. Most myxozoans are in the class Myxosporea and, for those whose life cycle has been elucidated, it involves alternation between two hosts, namely a vertebrate, usually a fish, and an invertebrate, currently known to be annelids [14–16]. Transmission between hosts is achieved by morphologically disparate waterborne spore stages: actinospores and myxospores.

All cnidarians are characterized by complex stinging organelles known as cnidae (cnidocysts) or as polar capsules in myxozoans. Free-living cnidarians have numerous cnidae the most studied of which function as an ultrafast toxin injection system, which paralyzes prey or deters predators [17–19]. Myxozoans have between one and approximately a dozen polar capsules, depending on life stage and genus, and are thought to use these to mechanically anchor to a host and initiate the infection process. Like cnidae, polar capsules contain a tightly coiled, long, inverted tubule known as a polar filament or, more accurately, a polar tubule. When activated, the tubule is ejected rapidly by eversion to anchor the spore to the host and, at least in some cases, contracts to pull the spore to the host [20, 21]. The amoeboid sporoplasm contained within the spore migrates to the target host tissue, where it proliferates and generates the next spore-producing stage [22–24]. Yet, despite the accumulation of epidemiological data about myxozoans [25] and the importance of the polar capsules for the initiation of infection, little is known about the specific functions of these organelles. Herein, we present a detailed analysis of the dynamics of the polar tubule discharge process in three *Myxobolus* species. We demonstrate that myxospore polar tubules variously perform a broader range of functions than previously appreciated, including ultrafast contraction and delivery of the capsule content.

## Methods

### Parasites and myxospores isolation

All three *Myxobolus* species were obtained from freshly caught fish. *Myxobolus klamathellus* was from a blue chub (*Gila coerulea*) caught in Klamath Lake, Oregon, USA, in November 2012. *Myxobolus shantungensis* was from a common carp (*Cyprinus carpio*) caught in the Willamette River, Oregon, USA, in 2013. Both of these myxozoans formed 5–10 mm diameter cysts from which myxospores were aspirated directly, and washed by centrifuging at low speed in PBS, then DDW, to remove host cells, then spore pellets were dried at ~ 40 °C in a Savant SpeedVac. *Myxobolus cerebralis* myxospores were obtained from juvenile rainbow trout exposed to laboratory cultures of infected *Tubifex tubifex* worms in 2015. Head cartilage was dissected out manually then homogenized with small volumes of DDW, filtered through 70 μm then 20 μm nylon mesh to concentrate spores, which were washed by centrifuging in DDW, before drying in a SpeedVac. All three spore types were rehydrated in DDW or 10 mM CaCl_2_ prior to the polar tubule experiments.

### Polar capsules labeling and activation

*Myxobolus* myxospore polar capsules were color-labeled using a solution of 0.05 M toluidine blue in 0.5 M NaCl for 10 min at room temperature. Spores were then spun down and re-suspended in DDW. Discharge was induced by adding 0.1 M NaOH.

### Image and movie processing

The polar capsule discharge process was recorded using an Andor Neo sCMOS black and white camera attached to a Nikon TI inverted epi-fluorescent microscope at an average rate of 200 frames per second, and resolution of 0.9 μm/pixel. Movies were then analyzed using Nikon’s image analysis program NIS-Elements version 3.22.15. The size of the polar capsule and the length of the elongating tubule were measured, and relative dye load was obtained from image pixel intensity throughout the discharge process. Color video clips were recorded using a Nikon DS-Fi1c camera (average 12 frames per second, and resolution 0.4 μm/pixel), mounted on a Nikon Eclipse 90i confocal microscope.

### Electron microscopy

Myxospores were attached to 0.01 % poly-D-lysine coated slides, and polar capsules were activated by 0.1 M NaOH. Thereafter, the samples were fixed in 2 % glutaraldehyde and 1 % paraformaldehyde in 0.2 M sodium cacodylate buffer (pH 7.4) for 2 h at 4 °C, then washed in 0.1 M cacodylate buffer and post-fixed in 1 % osmium tetroxide with the same buffer for 30 min at room temperature. Samples were dehydrated in an ascending ethanol series up to 100 %, transferred to 100 % acetone, air dried, coated by either gold or carbon and examined using Zeiss Sigma HD SEM.

## Results and discussion

**Fig. 1:**
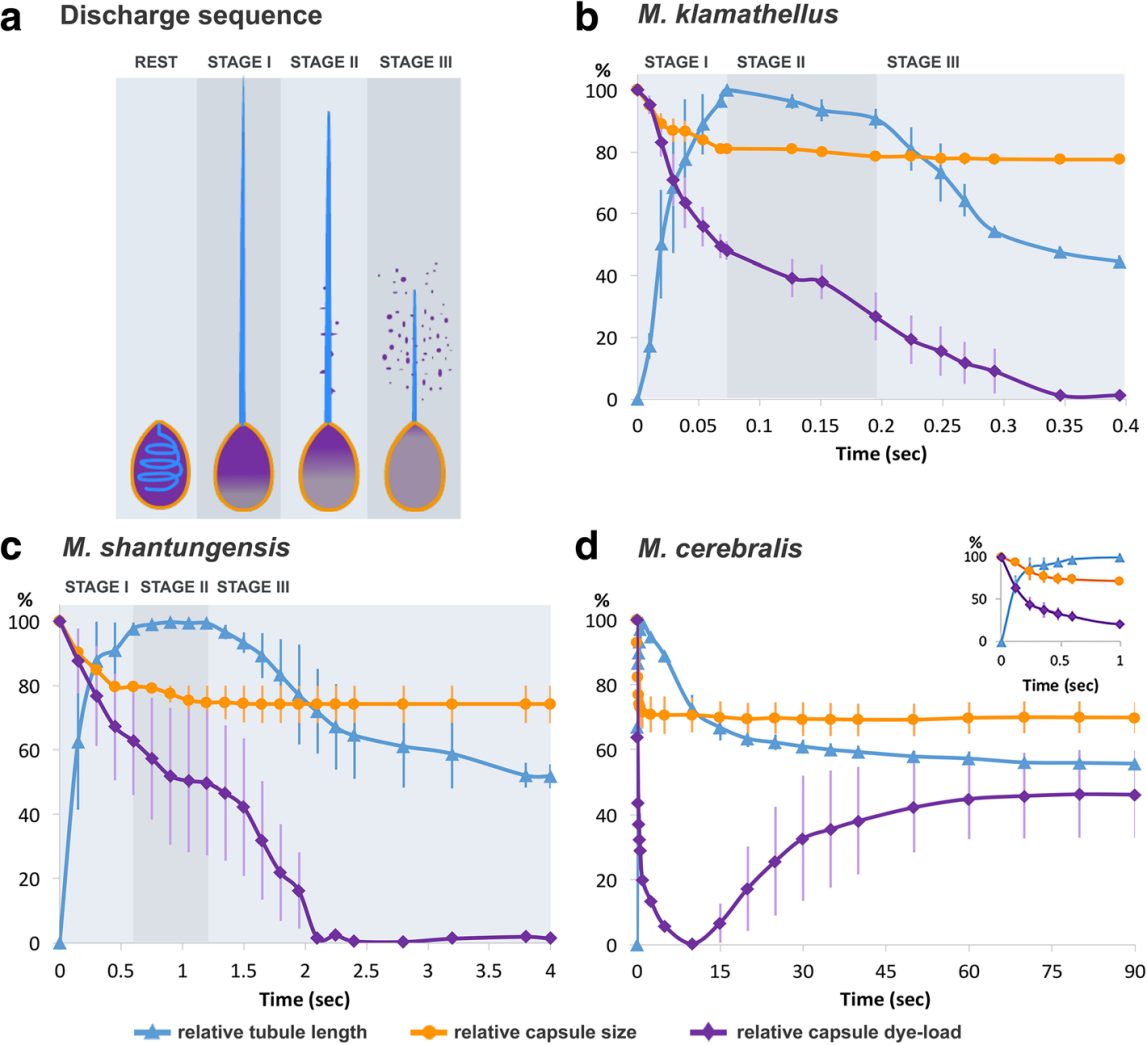
Polar capsule discharge dynamics. **a** Schematic representation of the discharge process in *Myxobolus klamathellus* (not to scale). From left to right: capsule at rest, with tubule folded inside; Stage I, capsule after discharge, with tubule fully extended; Stage II, tubule contraction and dye injection begins; Stage III, tubule fully contracted and all the dye expelled to the environment. **b** Graph of the discharge process in *Myxobolus klamathellus* myxospore. **c** Discharge in *Myxobolus shantungensis* myxospore. **d** Discharge in *Myxobolus cerebralis* myxospore. The Inset shows higher temporal resolution of the first second of the discharge process. Each graph shows the relative tubule length, relative capsule size and relative dye load within the capsule (all expressed as % from the maximum value) throughout the discharge process. Note the different time scales in **b**, **c** and **d**. Each curve is an average result obtained from three capsules, with the standard error of the mean

To study the function of the polar capsules, we compared three *Myxobolus* species: *M. klamathellus*, that infects epithelial tissues in kidney and skin in blue chub (Cyprinidae) [26], *M. shantungensis* that causes severe cartilage and soft tissue cyst development in common carp (Cyprinidae) [27] and *M. cerebralis*, the causative agent of whirling disease in trout (Salmonidae). We used toluidine blue to color-label the highly concentrated anionic poly-γ-glutamate content of the polar capsules [28, 29], triggered the discharge process with NaOH, and recorded it with a high-speed video camera to follow dye release by the ejected tubule. The dye allowed us to visualize the ejection dynamics (Fig. 1) and to test, for the first time, if the tubule acts as an injector similar to cnidae.

The most rapid discharge process, taking about 0.35 s, was seen in *M. klamathellus* and can be separated into three stages (Fig. 1b). In stage I (0.07 s), the tubule everted and filled with dye until it reached full extension (mean length ± SE, 93.0 ± 14.9 μm, *n* = 10), while capsule size decreases by ~ 20 %. In stage II (0.13 s), the tubule gradually contracted, decreasing in length by ~ 10 % and dye escaped through lateral openings in the proximal half of the tubule. In stage III (0.15 s), the tubule contracted rapidly to about half its extended length, acting like a pressure piston to expel the remaining dye content (Additional file 1: Video 1, and Additional file 2: Video 2). We found that in ~ 95 % of the activated spores (*n* = 80) the discharge of the first capsule was completed before the second capsule started its release (Additional file 1: Video 1). This finding may indicate that the two capsules have different sensitivity thresholds to prevent simultaneous triggering. We also found that if a tubule anchored after firing, the subsequent contraction process pulled the spore toward the anchor point (Additional file 3: Video 3).



**Video 1** Discharge of *Myxobolus klamathellus* polar capsules. Note the tubule contraction, and that injection occurs from the proximal half of the tubule. A red circle indicates a capsule that is about to discharge. *Scale*-*bar*: 14 μm.

**Video 2** Close-up of one of the *Myxobolus klamathellus* discharge polar Video 1. A red circle indicates the capsule that is about to discharge. *Scale*-*bar* 5 μm.

**Video 3** *Myxobolus klamathellus* close-up showing the anchoring and pulling ability of the extended tubule. A red circle indicates the capsule that is about to discharge, arrow indicates anchorage point, arrowhead indicates a tubule. *Scale*-*bar*: 5 μm.


**Fig. 2:**
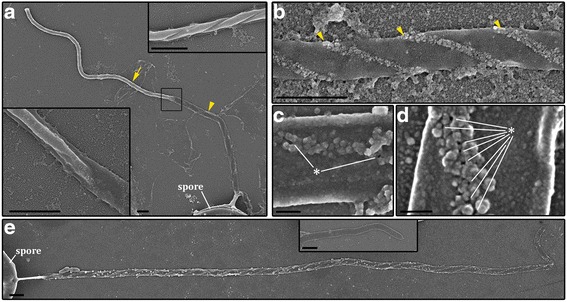
Morphological properties of *Myxobolus klamathellus* myxospore polar tubule as analyzed by SEM. **a** Overview of a tubule during the discharge process. The un-everted distal portion of the tubule (*arrow*) is still contained within the proximal everted portion (*arrowhead*). Top inset: enlargement of the un-everted portion (from another tubule) showing how the tubule is coiled before deployment. Bottom inset: enlargement showing the transition point between the two portions of the tubule shown in **a**. **b**-**d** Increasingly higher magnification views of the double spiral pattern observed on the outer surface of the tubule (*arrowheads*). Note the visible openings in **c** and **d** (*asterisks*). **e** Overview of a completely everted tubule demonstrating that the spiral pattern is thicker and more prominent in the proximal half. The pattern gradually disappears toward the tip. Inset shows complete lack of the pattern at the distal tip of a different tubule. **a**, **b** and **e** are gold-coated samples, **c** and **d** are carbon-coated. *Scale*-*bars*: 1 μm; **c**, 0.2 μm; **d**, 0.1 μm



The *M. shantungensis* myxospores exhibited similar discharge dynamics, but the process lasted longer (~2 s) and the content release occurred more toward the distal end of the extended tubule rather than at the proximal half (Fig. 1c, Additional file 4: Video 4). In both cases little dye escaped the tubules prior to their full extension. We hypothesize that this dye release delay is caused by the inability of the tubule pores to open earlier, and that two mechanisms are plausible: a minimum tension must be developed along the tubule wall to open its pores, and/or while the uneverted part of the tubule remains in contact with the external wall of the everting tubule (Fig. 2a) the pores are prevented from opening until it passes through. Another possible explanation is that as long as the tubule is not fully released, hydraulic resistance is too high for the dye, which exits the pores only when the tubule is fully everted.

**Video 4** Discharge of *Myxobolus shantungensis* polar capsule. Note tubule contraction, and that injection occurs from the distal half of the tubule. A red circle indicates the capsule that is about to discharge. *Scale*-*bar*: 5 μm.


Different discharge dynamics were seen in *M. cerebralis* myxospores. The eversion process was much slower, taking more than 10 s to be completed (Fig. 1d), similar to what has been found in the fish-infecting actinospores [21]. Surprisingly, no dye was released from the tubule in this species, suggesting that it had no openings. The dye was pushed forward into the tubule and then back to the capsule, showing the piston-like effect due to the elastic contraction (Additional file 5: Video 5). This last observation implies that tubule contraction does not require pressure release via tubule opening. Thus, polar tubules of *Myxobolus* myxospore display high functional diversity, exhibiting different contraction dynamics and delivery capabilities, which might be a result of adaptation to diverse invertebrate hosts or infection routes.



**Video 5** Discharge of *Myxobolus cerebralis* polar capsule. Note the movement of the dye from the capsule to the tubule and back again. A red circle indicates the capsule that is about to discharge, a black arrow indicates dye returning to the capsule and a red arrow indicates movement in the tubule. *Scale*-*bar*: 5 μm.


The observed manner of content delivery in these myxozoans is atypical for cnidae in free-living cnidarians. In all previous studies of injecting cnidae, the injection process has resembled that of hypodermic needles, with cnida content exiting mainly through the tubule tip and, in some cases, through additional lateral openings [30–32]. Here we present evidence of a different apparatus, whereby the content is released only from lateral openings and the tubule tip is sealed. This type of delivery could maximize the contact area between the released compounds and the host’s surface, supporting the hypothesis of an adhesive or digestive function. A recent transcriptome analysis of *Myxobolus pendula* identified putative toxin proteins, similar to those found in cnidae [33]; however, further analysis is needed to determine whether myxozoan capsules contain toxins and, if so, their possible role.

Despite the broad range of cnidae structures and functions in free-living cnidarians, none have been shown to exhibit contracting tubules or elasticity, suggesting that the structural composition of at least some myxozoan tubules [21] is inherently different from their free-living cousins. Interestingly, tubule contraction had previously been reported in cnidae-like structures of dinoflagellates [34]. This finding adds to other similarities between cnidarians and dinoflagellates that bear cnidae-like structures [34–36]. Several hypotheses have been proposed to account for these remarkable similarities, including convergent evolution, common ancestry and horizontal gene transfer, perhaps as part of symbiotic relationship between a dinoflagellate and an archaic cnidarian [14, 34, 37, 38].

Next we focused on characterization of the tubule ultrastructure throughout the discharge process by means of scanning electron microscopy (SEM) of *M. klamathellus* (Fig. 2). During tubule eversion and extension, the remaining inverted tubule was seen within the elongating tubule (Fig. 2a). After complete eversion, a double spiral pattern of amorphous globular structures was observed on the outer surface (Fig. 2b). Higher magnification revealed the presence of openings in the tubule wall, positioned among the globular structures (Fig. 2c, d). The material composing the helical pattern was much thicker and denser on the proximal half of the tubule and could be completely absent distally (Fig. 2e insert). Interestingly this distribution correlated with the observed spatial pattern of dye release, suggesting that it may increase polar tubule adhesion. Another possible benefit of the globular structures is the prevention of pores clogging in host epithelial environments, where mucus or other barrier defense compounds may be present.

Spiral patterns are found on cnidae tubules of most cnidarian groups. In free-living Cnidaria, there are three helical rows that may be armed with different types of spines [30]. However the unique parasitic hydrozoan *Polypodium hydriforme* has only two rows [39]. Our finding of double-helical symmetry is consistent with the double-folded pattern seen in TEM analysis of undischarged capsules of *M. cerebralis* [40], and provides additional support for the suggestion that the helical pattern results from the folding of the inverted tubule inside the capsule [39]. Helical shapes are relatively flexible and distribute normal and shear stresses in both radial and axial directions. Therefore, we suggest that a spiral distribution of openings, as opposed to a ring-like or linear arrangement, would allow the tubule to better withstand the stresses caused by the rapid and dynamic mechanical changes during firing.

The structures that constitute the spiral pattern can vary from simple amorphous blobs to complex spines as in free-living cnidarians and, as we show here, may also contain openings. This could imply a conserved developmental scheme for tubules across all Cnidaria, with species-specific morphological and functional adaptations arising from less conserved developmental pathways. Hence, while the formation of the primary pattern could be a non-adaptive by product of development, structures and openings may be regulated by an evolvable module [41] that enables variation in tubule properties without changing the underlying developmental program.

## Conclusions

We show in spores of several *Myxobolus* species that the polar tubules of these parasitic cnidarians have diverse functions and discharge dynamics. In addition to anchoring to the host, and pulling the spore toward it, tubules may also serve as a delivery device that releases the capsule content into the immediate host environment. We speculate that the released substances may facilitate host adhesion or penetration.

## Abbreviations

SEM: Scanning electron microscopy
